# Combination of temozolomide with immunocytokine F16–IL2 for the treatment of glioblastoma

**DOI:** 10.1038/sj.bjc.6605832

**Published:** 2010-08-24

**Authors:** M Pedretti, C Verpelli, J Mårlind, G Bertani, C Sala, D Neri, L Bello

**Affiliations:** 1 Institute of Pharmaceutical Sciences, Department of Chemistry and Applied Biosciences, ETH-Swiss Federal Institute of Technology Zurich, Wolfgang-Pauli-Strasse 10, 8093 Zurich, Switzerland; 2 Institute of Neurosciences, CNR, Via Vanvitelli 32, 20129 Milan, Italy; 3 Department of Neurological Sciences, University of Milan, IRCCS Ospedale Maggiore, Via Francesco Sforza 35, 20122 Milan, Italy

**Keywords:** glioblastoma, A1 domain of tenascin-C, interleukin-2, immunocytokines, temozolomide

## Abstract

**Background:**

Glioblastoma patients are still not cured by the treatments available at the moment. We investigated the therapeutic properties of temozolomide in combination with F16–IL2, a clinical-stage immunocytokine consisting of human interleukin (IL)-2 fused to the human antibody F16, specific to the A1 domain of tenascin-C.

**Methods:**

We conducted three preclinical therapy studies, using subcutaneous and intracranial U87MG glioblastoma tumours xenografted in BALB/c nude mice. The same therapeutic schedule was used, consisting of five total administrations every third day, of 0.525 mg temozolomide, 20 *μ*g F16–IL2, the combination, or the control solution.

**Results:**

Immunohistochemical analysis of U87MG xenografts and of human glioblastoma specimens showed selective tumour staining of F16. A quantitative biodistribution confirmed the preferential tumour accumulation of radiolabelled F16–IL2. In the study with subcutaneous xenografts, the combination of F16–IL2 with temozolomide induced complete remission of the animals, which remained tumour free for over 160 days. The same treatment led to a consistent size reduction of intracranial xenografts and to a longer survival of animals. The immunocytokine promoted the recruitment of leukocytes into tumours of both models.

**Conclusion:**

The combined use of temozolomide with F16–IL2 deserves clinical investigations, which will be facilitated by the excellent safety profile in cynomolgus monkeys, and by the fact that F16–IL2 is in clinical trials in patients with cancer.

Central nervous system tumours rank first among neoplasia types for the average years of life lost (Burnet *et al*, 2005). Approximately 13 000 deaths and 18 000 new cases of central nervous system tumours occur annually in the United States (CBTRUS Statistical report, 2006). Mortality rates are generally similar to incidence rates in most geographical areas (Ferlay *et al*, 2000).

The term ‘glioma’ refers to tumours of glial cell origin and includes astrocytomas, oligodendrogliomas, ependimomas, and mixed gliomas (Kleihues and Cavenee, 2000). They account for more than 70% of all brain tumours and their prognosis is very poor. Glioblastoma is the most frequent (65% of all gliomas) and also the most malignant histological type (Ohgaki *et al*, 2004).

The prognosis of glioblastoma continues to be dismal in spite of progress made in the molecular characterisation of the most frequent genetic alterations of the disease (Parsons *et al*, 2008). Even state-of-the art multimodality treatments, although capable of substantially extending life expectancy, are not curative (Stupp *et al*, 2005). The current standard of care for patients with glioblastoma includes surgery, radiotherapy, and concurrent and adjuvant temozolomide (Stupp *et al*, 2005). Temozolomide is an oral alkylating drug that has demonstrated antitumour activity as a single agent in the treatment of recurrent glioma (Newlands *et al*, 1997; Yung *et al*, 2000; Stupp *et al*, 2001). Temozolomide is indicated for newly diagnosed glioblastoma patients in association with radiotherapy in the postsurgical period (75 mg m^−2^ p.o. daily for 6 weeks, during a focal radiotherapy taking place five times a week for 6 weeks, for a total dose of 60 Gy), and alone during the maintenance period (150–200 mg m^−2^ p.o. on days 1–5, every 28 days for six cycles). Nevertheless, this treatment modality yields a median survival benefit of 2.5 months, compared with adjuvant radiotherapy alone (Stupp *et al*, 2005).

Conventional cytotoxic therapies of cancer often do not discriminate between tumour and normal tissues. To achieve therapeutically relevant concentrations in the tumour mass, large drug doses have to be administered to the patient, leading to a poor therapeutic index and unacceptable toxicities to healthy tissues (Bosslet *et al*, 1998). The selective delivery of therapeutic agents to the tumour site, using antibodies directed against tumour-associated antigens, represents a promising strategy to overcome the disadvantages of conventional cancer therapies (Adams and Weiner, 2005; Carter, 2006; Schrama *et al*, 2006). Antigens that are expressed around the tumour neovasculature are especially attractive targets for antibody-based pharmacodelivery applications because of their inherent accessibility for blood-borne agents and because angiogenesis is a characteristic feature of virtually all aggressive solid tumours (Thorpe, 2004; Neri and Bicknell, 2005; Schliemann and Neri, 2007). Our group has demonstrated the possibility of delivering bioactive agents to the subendothelial extracellular matrix using monoclonal antibodies specific to splice isoforms of fibronectin and tenascin-C (Birchler *et al*, 1999; Carnemolla *et al*, 2002; Halin *et al*, 2002, 2003; Borsi *et al*, 2003; Ebbinghaus *et al*, 2005; Neri and Bicknell, 2005; Tijink *et al*, 2006; Kaspar *et al*, 2007). In particular, encouraging results obtained with derivatives of the human monoclonal antibodies L19 (specific to the extradomain B of fibronectin) and F16 (specific to the extradomain A1 of tenascin-C) have led to the clinical development of five immunocytokines and radioimmunoconjugates (Carnemolla *et al*, 2002; Halin *et al*, 2002, 2003; Borsi *et al*, 2003; Ebbinghaus *et al*, 2005; Menrad and Menssen, 2005; Schliemann and Neri, 2007; Mårlind *et al*, 2008).

Microvascular proliferation is a characteristic feature of glioblastoma (Kleihues and Cavenee, 2000; Castellani *et al*, 2002). An overexpression of the extradomain B of fibronectin in high-grade gliomas has been established and the monoclonal antibody L19 has been shown to target glioblastoma in patients (Castellani *et al*, 1994, 2002; Santimaria *et al*, 2003). Furthermore, radiolabelled preparations of monoclonal antibodies specific to the A1 or the D domain of tenascin-C have been investigated for the radioimmunotherapy of patients with glioblastoma (Riva *et al*, 2000; Paganelli *et al*, 2006; Zalutsky *et al*, 2007, 2008).

In this study, we have explored the possibility of using immunocytokine F16–IL2 in combination with temozolomide for the therapy of experimental murine models of glioblastoma. F16–IL2 is a noncovalent homodimeric immunocytokine consisting of the human proinflammatory cytokine interleukin (IL)-2 fused to the human antibody fragment scFv(F16). Both F16 and F16-IL2 have been shown to intensely stain aggressive cancer types and to preferentially accumulate at the tumour site following intravenous administration (Brack *et al*, 2006; Mårlind *et al*, 2008). F16–IL2 has recently been shown to mediate an anticancer effect, which can be potentiated by coadministration of taxanes or adriamycins, leading to the initiation of two phase Ib clinical studies in patients with metastatic breast or ovarian cancer (F16–IL2+doxorubicin), or breast or lung cancer (F16–IL2+paclitaxel). Tumour-targeting immunocytokines based on IL2 have previously been demonstrated to mediate the infiltration of immune cells into the tumour mass, with natural killer (NK) cells as the main mediator of therapeutic activity (Reisfeld and Gillies, 1996; Carnemolla *et al*, 2002; Mårlind *et al*, 2008; Schliemann *et al*, 2009). These properties may be beneficial in the context of glioblastoma therapy (Dunne *et al*, 2001; Albertsson *et al*, 2003). In our experiments, F16–IL2 was found to potentiate the therapeutic action of temozolomide in nude mice bearing subcutaneous and orthotopic human glioblastoma xenografts.

## Materials and methods

### Cell lines and animals

Human subcutaneous and orthotopic glioblastoma xenografts were obtained by injection of U87MG human glioblastoma cells (ATCC Nr: HTB-14). This cell line was cultured in MEM (Invitrogen, Basel, Switzerland), supplemented with 10% fetal calf serum (Invitrogen), 2 mM L-glutamine, 1 mM sodium pyruvate, and 100 U mL^−1^ ampicillin, and incubated at 37°C in 5% CO_2_. Animal experiments with subcutaneous glioblastoma xenografts were conducted in female BALB/c nude mice (Charles River Laboratories, Sulzfeld, Germany) under a project licence granted by the Veterinäramt des Kantons Zürich (198/2005), whereas orthotopic glioblastoma therapy experiments were conducted in male BALB/c nude mice (Charles River Laboratories), according to the University of Milan animal facility rules.

### Antibodies and therapeutic agents

The L19 antibody, specific to the extradomain B of fibronectin, the F16 antibody, specific to the extradomain A1 of tenascin-C, and the preparation and characterisation of the F16–IL2 fusion protein with human interleukin-2 have been described before (Pini *et al*, 1998; Neri and Bicknell, 2005; Brack *et al*, 2006; Mårlind *et al*, 2008) Temozolomide (ABCR GmbH & Co. KG, Karlsruhe, Germany) was dissolved in a saline solution (H_2_O 0.9% NaCl) containing 10% dimethyl sulfoxide (DMSO).

### Immunohistochemistry on human glioblastoma samples and glioblastoma xenografts

Surgically resected human glioblastoma tissues and U87MG subcutaneously xenografted glioblastomas were freshly frozen in OCT (optimal cryotemperature) medium as described (Borsi *et al*, 2002; Castellani *et al*, 2002) and stored at −80°C before being processed.

For immunohistochemical procedures, F16 and L19 antibodies were used in biotinylated small immunoprotein (SIP) format (Borsi *et al*, 2002; Brack *et al*, 2006). Aliquots of antibodies were prepared from a single batch, stored at 4°C, and used only once, thus contributing to excellent reproducibility of immunohistochemical results.

Tissue sections of 10 *μ*m thickness were treated with ice-cold acetone, rehydrated in Tris buffer solution (50 mM Tris, 100 mM NaCl, 0.001% Aprotinin, pH 7.4), and blocked with Tris buffer solution 20% fetal calf serum. Biotinylated SIP(F16) and SIP(L19) were added onto the sections in a final concentration of 2 *μ*g ml^−1^ and detected using a streptavidin–alkaline phosphatase complex (Biospa, Milano, Italy) as described (Borsi *et al*, 2002; Castellani *et al*, 2002). Fast Red (Tablets Set, Sigma-Aldrich Chemie GmbH, Steinheim, Germany) was used as phosphatase substrate and sections were counterstained with Gill's haematoxylin no. 2 (Sigma-Aldrich Chemie GmbH). For every immunohistochemical experiment, a negative control was used by omitting the primary antibody.

The optic microscope Zeiss Axiovert S100TV (Carl Zeiss MicroImaging GmbH, Jena, Germany), at 5 × , 10 × , and 20 × magnifications, and the Zeiss Axiovision Release 4 acquisition software were used to evaluate the expression of the A1 domain of tenascin-C and the extradomain B of fibronectin, as revealed by staining using F16 and L19 antibodies, respectively.

### Biodistribution experiment

The *in vivo* targeting performance of F16-IL2 was evaluated by quantitative biodistribution analysis (Carnemolla *et al*, 2002). Female BALB/c nude mice bearing subcutaneous U87MG tumours (obtained by a s.c. flank injection of 5 × 10^6^ U87 cells) were grouped (*n*=5/group) when tumours were clearly palpable (volume of ca 200 mm^3^) and injected i.v. into the lateral tail vein with radioiodinated F16–IL2. Antibody immunoreactivity after labelling was evaluated by loading a sample of radiolabelled F16–IL2 onto TNC-A1-Sepharose resin, followed by radioactive counting of the flow-through and eluate fractions. Immunoreactivity, defined as the ratio between the counts of the eluted protein and the sum of the counts of the eluted and flow-through fractions, was 84%. Mice were killed 24 h after injection of F16–IL2 (10 *μ*g, 3.6 *μ*Ci per mouse), organs were weighed, and radioactivity was counted with a Packard Cobra gamma counter (GMI Inc, Ramsey, MN, USA). The radioactivity content of representative organs was expressed as the percentage of the injected dose per gram of tissue (%ID g^−1^).

### Subcutaneous glioblastoma mouse model

Subcutaneous glioblastoma-bearing mice were obtained by a s.c. flank injection of 5 × 10^6^ U87MG cells in 8-week-old female BALB/c nude mice. Twelve days after tumour cell implantation, when tumours had reached an average size of 300 mm^3^, mice were staged to maximise uniformity among the groups (*n*=5/group). One group was injected i.v. (lateral tail vein) with 20 *μ*g of F16–IL2 (corresponding to 6.6 *μ*g of IL-2) in a total volume of 100 *μ*l PBS (phosphate-buffered saline) solution, one was injected i.p. with 0.525 mg of temozolomide (corresponding to 75 mg m^−2^) in a total volume of 150 *μ*l saline 10% DMSO, a third group received both the i.v. injection of 20 *μ*g F16–IL2 and the i.p. of 0.525 mg temozolomide. Finally, the control group was injected i.p. with 150 *μ*l of saline 10% DMSO. Five total administrations were performed on days 12, 15, 18, 21, and 24. Using a conversion factor based on body surface (Reagan-Shaw *et al*, 2008), the dose of temozolomide used in this study corresponded to a human dose of 75 mg m^−2^ for each injection (Stupp *et al*, 2005). The cumulative dose of temozolomide per mouse was lower than the LD_10_ (dose lethal to 10% of treated animals) for both subcutaneous and orthotopic glioblastoma mouse models (Friedman *et al*, 2000).

Animals were monitored and weighted daily; tumours were measured with a digital caliper three times a week. Tumour volume was estimated using the formula volume=length × width^2^/2. Mice were killed when tumours approached a volume of 3000 mm^3^ or when tumours turned necrotic and bled, according to Swiss regulations. No animals had to be killed because of therapy-derived toxicities.

### Orthotopic glioblastoma mouse model

Orthotopic glioblastoma-bearing mice were obtained by an intracranial implantation of 5 × 10^4^ U87MG cells in 6-week-old male BALB/c nude mice (Bello *et al*, 2004). Twelve days after tumour cell implantation, mice were randomly divided into four therapeutic groups (*n*=10/group). Schedule and doses of therapeutics were the same as for the therapy with subcutaneous glioblastoma-bearing mice.

Mice were monitored daily to detect any signs of neurological suffering from tumour growth or from toxicity effects of the therapy. All therapeutic groups were killed 25 days after therapy beginning. Brains were removed and the hemisphere containing the tumour and the contralateral one were separately snap-frozen and stored at −80°C. Tumours were measured by thawing the corresponding hemisphere and by sectioning the tissue when necessary. Tumour volume was estimated using the formula volume=length × width^2^/2. The healthy hemisphere was taken as control.

We repeated the therapy with intracranial U87MG xenografted mice, comparing the four therapeutic groups on a survival basis. Orthotopic glioblastoma-bearing mice were obtained by an intracranial implantation of 5 × 10^4^ U87MG cells in 6-week-old male BALB/c nude mice (Bello *et al*, 2004). Twelve days after tumour cell implantation, mice were randomly divided into four therapeutic groups (*n*=8/group and *n*=9 for the temozolomide+F16–IL2 combination group). Schedule and doses of therapeutics were the same as for the previous therapies. Mice were monitored daily and killed at the first appearance of neurological damage from tumour growth or on detection of any signs of suffering from therapy-related toxicity. Kaplan–Meier survival curves were drawn.

### Assessment of immune effector cell infiltration in subcutaneous and intracranial glioblastoma xenografts, and in normal organs following treatment

To evaluate the role of inflammatory cell responses, BALB/c nude mice bearing s.c. or i.c. U87MG tumours (*n*=3/therapeutic group) were treated on days 12, 15, and 18 after tumour cell implantation with F16–IL2 (i.v.), temozolomide (i.p.), F16–IL2 (i.v.) plus temozolomide (i.p.), or saline 10% DMSO solution (i.p.). Mice were killed 24 h after the third injection. Tumours were excised and snap-frozen in OCT medium, and liver and kidneys were fixated in 4% formalin and embedded in paraffin according to standard procedures.

The immunofluorescent staining of subcutaneous and intracranial tumour sections was performed using antibodies against the following antigens: F4/80 (rat antimouse F4/80, clone A3-1, AbCam, Cambridge, UK) for the detection of tumour-infiltrating macrophages, asialo GM1 (rabbit antiasialo GM1, Wako Pure Chemical Industries Ltd, Osaka, Japan) for NK cells, and CD45 (rat antimouse CD45, BD Biosciences Pharmingen, Allschwil, Switzerland) for leukocytes. In all cases, CD31 staining (rabbit or rat antimouse CD31, BD Biosciences Pharmingen) was performed to identify vascular structures.

Frozen tumour sections of 10 *μ*m thickness were treated with ice-cold acetone, blocked with PBS 10% donkey serum+10% goat serum, incubated with primary antibodies (in a PBS 12% bovine serum albumin (BSA) solution), and detected using fluorescent Alexa 488- or Alexa 594-coupled secondary antibodies (donkey antirat or goat antirabbit IgG, BD Biosciences Pharmingen) in a PBS 12% BSA solution. The microscope Zeiss Axioskop 2 mot plus with the fluorescence lamp HXP 120 Kubler Codix (Carl Zeiss MicroImaging GmbH), and the acquisition software Zeiss Axiovision Release 4 were used for analysis.

Paraffin-embedded liver and kidney sections from mice of the different therapeutic groups were baked overnight at 60°C and deparaffinised according to standard procedures. Antigen retrieval was obtained by microwave warming in 8.2% trisodium citrate (0.1 M)+1.8% Citric Acid (0.1 M) solution. Sections were blocked with PBSTT (PBS 0.5% Tween+0.1% Triton-X) 10% BSA, incubated first with the primary antibody rat antimouse CD45 (clone 30-F11, BD Biosciences Pharmingen), then with the secondary antibody biotinylated mouse antirat IgG_2_ (clone G15-337, BD Biosciences Pharmingen), followed by Streptavidin Alexa 488-coupled+4'-6-diamidino-2-phenylindole. Glioblastoma tumours from the same mice were used as positive control.

In each tissue section, staining was quantified in three representative microscopic images using ImageJ software (http://rsb.info.nih.gov/ij/) and expressed as a percentage of measurement area.

### *Ex vivo* detection of the F16–IL2 fusion protein in subcutaneous and intracranial glioblastoma xenografts following treatment

The *in vivo* localisation of the F16–IL2 fusion protein within the tumour mass was investigated in subcutaneous and intracranial glioblastoma xenografts collected from BALB/c nude mice (*n*=3/therapeutic group), which were treated on days 12, 15, and 18 (day 0=tumour cell implantation) with F16–IL2 or with the combination of F16–IL2 and temozolomide, and killed 24 h after the third drug administration.

A rat antihuman IL-2 antibody (eBioscience Inc., San Diego, CA, USA) was used to detect the F16–IL2 fusion protein within the tumour mass, and the CD31 staining (rabbit anti-mouse CD31, BD Biosciences Pharmingen) served to identify vascular structures. Immunofluorescent staining and analysis of results were performed in the same way as for the immune cell infiltration study.

The specific binding of the rat antihuman IL-2 antibody to the F16–IL2 immunocytokine was validated by enzyme-linked immunosorbent assay. We loaded the biotinylated tenascin-C A1 antigen (10^−6^ M) on streptavidin wells (A–H), followed by 5 *μ*g ml^−1^ of F16–IL2 in PBS 2% milk in wells A–C, and 5 *μ*g ml^−1^ of F8-IL2 in wells D–F as irrelevant antibody; wells G–H served as negative control without primary antibody. The rat antihuman IL-2 antibody (eBioscience Inc; diluted 1 : 1000) was used as secondary antibody (wells A–H), followed by the goat antirat IgG-HRP (eBioscience Inc.; diluted 1 : 1000), the POD substrate (Roche Diagnostic, Rotkreuz, Switzerland), and the H_2_SO_4_ 1 M solution to complete the reaction.

### Assessment of apoptosis and proliferation in subcutaneous glioblastoma xenografts following treatment

The analysis of apoptosis and proliferation induced by the therapy was performed in subcutaneous glioblastoma xenografts collected from BALB/c nude mice (*n*=3/therapeutic group), which were treated on days 12, 15, and 18 (day 0=tumour cell implantation) with F16–IL2, temozolomide, the combination of F16–IL2 and temozolomide, or saline 10% DMSO solution, and killed 24 h after the third drug administration.

Fluorescent TUNEL (Terminal deoxynucleotidyl transferase dUTP nick end labeling) assays (Roche Diagnostic) were performed according to the manufacturer's instructions to detect apoptosis in tumours of the different therapeutic groups.

The same specimens were analysed by immunofluorescence to detect proliferation as revealed by the Ki67 antigen. Tumour sections were blocked first with PBS 10% donkey serum+10% goat serum, then with AffiniPure Fab fragment goat antimouse IgG (Jackson ImmunoResearch Laboratories Inc., West Grove, PA, USA) in a PBS 12% BSA solution. A monoclonal mouse antihuman Ki67 (clone B126.1, AbCam) and a rat antimouse CD31 (BD Biosciences Pharmingen) were used as primary antibodies, followed by Alexa 594-coupled donkey antirat IgG and Alexa 488-coupled goat antimouse IgG (BD Biosciences Pharmingen)+4'-6-diamidino-2-phenylindole in a PBS 12% BSA solution. The analysis and quantification of results were performed in the same manner as for the previous immunofluorescence experiments.

### Statistics

Comparisons of data of the efficacy study with intracranial xenografts and of the infiltration and apoptosis/proliferation studies were performed using a two-tailed Student's *t*-test. Survival analysis was conducted by Kaplan–Meier curves, and their comparison was determined by log-rank test. *P*-values of <0.05 were considered significant.

## Results

### Immunohistochemistry on human glioblastoma specimens and on mouse U87MG xenografts

**Figure 1:**
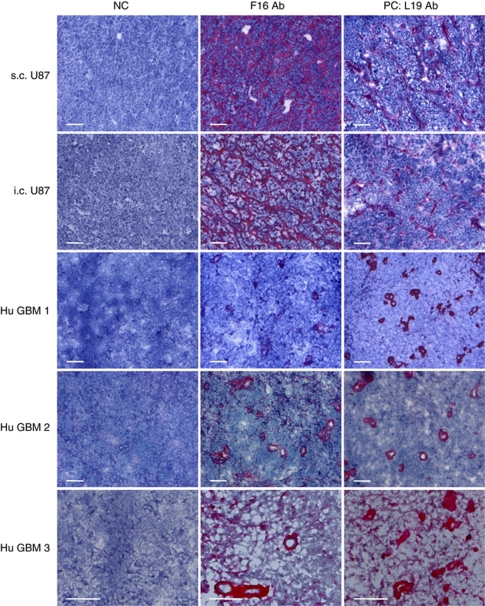
Immunohistochemical analysis of U87MG human glioblastoma xenografts and of human glioblastoma surgical specimens using the F16 antibody, specific to the extradomain A1 of tenascin-C, and the L19 antibody, specific to the extradomain B of fibronectin (serial tissue sections). Both antibodies stained tumour perivascular structures considerably. In negative controls (NC), the primary antibody was omitted. Scale bars indicate 100 *μ*m.

We assessed the expression of the A1 domain of tenascin-C and of the extradomain B of fibronectin (as positive control) in sections of human glioblastoma surgical specimens and of U87MG xenografts in nude mice. We used identical concentrations of F16 and L19 antibodies (Pedretti *et al*, 2009). F16 was found to strongly stain both experimental U87MG tumours and glioblastoma samples from patients, with patterns and intensities comparable to the ones of L19 (Figure 1), which had previously been reported to stain perivascular structures in high-grade gliomas (Castellani *et al*, 2002).

### Biodistribution study with radiolabelled F16–IL2

Nude mice bearing subcutaneous U87MG glioblastomas were injected i.v. with radioiodinated preparations of F16-IL2 to study the *in vivo* targeting performance by quantitative biodistribution analysis. The immunocytokine displayed a preferential accumulation in the tumour 24 h after injection (2.3% ID g^−1^), with a tumour-to-blood ratio of 11.5 and with excellent tumour-to-organ ratios (Supplementary Figure 1). In our previous experience, normal brain and muscle exhibited uptake levels at least 10 times lower than that of other organs (Berndorff *et al*, 2006; Spaeth *et al*, 2006).

### Therapeutic activity of F16–IL2 combined with temozolomide in subcutaneous and intracranial glioblastoma xenografts

We compared the therapeutic activity of F16–IL2 and of temozolomide (alone and in combination) in nude mice bearing subcutaneous U87MG tumours. Therapy was started 12 days after subcutaneous injection of U87MG cells, when tumours had reached an average size of 300 mm^3^. Temozolomide was administered five times, every third day, with i.p. injections of 0.525 mg in saline 10% DMSO. This dose is higher than the one used in previous therapy studies with the same agent (Chakravarti *et al*, 2006; Yamini *et al*, 2007), but is well below the LD_10_ of temozolomide in nude mice, reported to be equal to 411 mg m^−2^ administered i.p. daily for 5 days, and to 1200 mg m^−2^ administered i.p. once (Friedman *et al*, 2000). The dose of temozolomide used in this study corresponds to a human dose of 75 mg m^−2^ for each injection, in line with the standard 75 mg m^−2^ p.o. daily dose of temozolomide, administered to patients for 6 weeks during adjuvant radiation therapy (Stupp *et al*, 2005; Reagan-Shaw *et al*, 2008). F16–IL2 was administered i.v. at 20 *μ*g doses (corresponding to 6.6 *μ*g of IL2).

**Figure 2:**
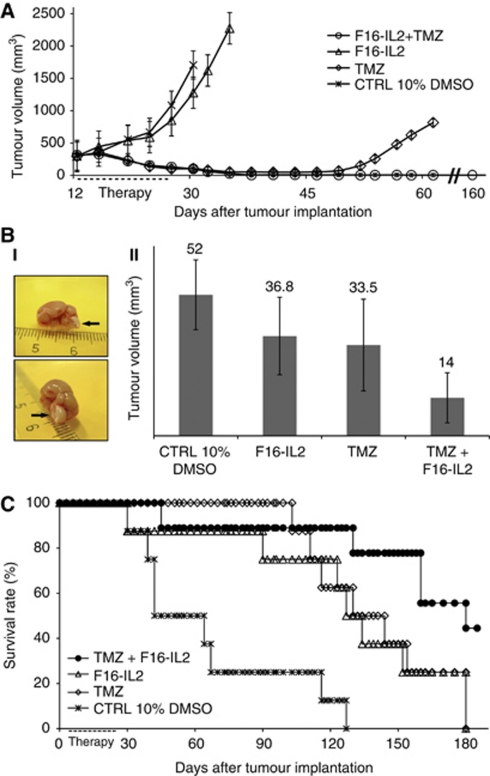
(**A**) Preclinical therapy study with subcutaneous U87MG human glioblastoma xenografts. The treatment regimen consisted of five total administrations, every third day, of temozolomide (0.525 mg, corresponding to 75 mg m^−2^) in a saline 10% dimethyl sulfoxide (DMSO) solution, F16–IL2 (20 *μ*g) in phosphate-buffered saline, a combination of F16–IL2 and temozolomide (same doses), or saline 10% DMSO solution. The combination therapy group exhibited the highest therapeutic benefit with a complete remission of the animals, which remained tumour free for over 160 days. (**B**) Preclinical therapy study, using intracranial U87MG human glioblastoma xenografts. The same therapeutic schedule of the subcutaneous study was used. The combination of F16–IL2 with temozolomide exhibited the highest therapeutic benefit. Pairwise comparisons between the combination therapy group and temozolomide alone (*P*=0.009), F16–IL2 alone (*P*=0.001), and the control group (*P*<0.001) were calculated using the Student's *t*-test and showed significant results. (**I**) Photograph of a mouse hemisphere with tumour, imaged from two sides. (**II**) Tumour volumes at day 25 from the start of treatment (13 days after the last drug administration), expressed as average mean±s.d. (**C**) Survival study using intracranial U87MG human glioblastoma xenografts, with the same therapeutic schedule of the previous subcutaneous and intracranial studies. Results indicate a longer survival for the combination treatment group (combo *vs* TMZ: *P*<0.002; combo *vs* F16–IL2: *P*<0.002; combo *vs* control: *P*<0.0001).

Monotherapy treatment with F16–IL2 led to a minor tumour growth retardation, compared with the control group of mice treated with saline 10% DMSO, whereas all mice in the temozolomide group exhibited a strong tumour regression by day 30 (Figure 2A). However, by day 45, tumours started to grow again in three out of five mice. By contrast, mice treated with the combination of F16–IL2 plus temozolomide exhibited a complete remission and remained tumour free for over 160 days.

The toxicity of pharmacological treatments in tumour-bearing mice is commonly evaluated by a constant monitoring of weight loss. In this study, mice of the control group did not exhibit weight loss, whereas mice of the temozolomide, F16–IL2, and F16–IL2 plus temozolomide treatment groups exhibited a comparable profile of weight loss, which was at all time points below 9% (Supplementary Figure 2A).

Encouraged by the tumour eradication obtained in subcutaneous xenografts, we studied the therapeutic performance of F16–IL2 and temozolomide in an intracranial model of glioblastoma, obtained by stereotactic injection of 5 × 10^4^ U87MG cells into nude mice. The treatment schedule and doses were as for the subcutaneous model, but all mice were killed on day 25 from the start of therapy to allow a comparative evaluation of tumour burden for the four treatment groups. This time point was chosen as the end of the experiment (i.e., after killing all mice), as the first animal in the control group started to show signs of neurological damage. Mice of the combination therapy (F16–IL2+temozolomide) exhibited the strongest therapeutic benefit, with an average tumour volume of 14 mm^3^, compared with those of temozolomide alone (33.5 mm^3^), F16–IL2 alone (36.8 mm^3^), and the control group (52 mm^3^). Pairwise comparisons between the combination therapy group and temozolomide alone (*P*=0.009), F16–IL2 alone (*P*=0.001), and the control group (*P*<0.001) were calculated using the two-tailed Student's *t*-test and showed significant results (Figure 2B). No signs of distress were observed in the animals during the entire treatment period.

We performed a further therapy experiment with intracranial glioblastoma-bearing mice to better evaluate the therapeutic benefit of F16–IL2 plus temozolomide on a survival basis. Four groups of mice were treated with the same doses and schedule as for the previous studies and were killed at the first appearance of neurological damage from tumour growth or on detection of any treatment-related toxicities. The Kaplan–Meier survival curve confirms the higher therapeutic benefit for the F16–IL2 plus temozolomide combination group (Figure 2C), with no augmented toxicity (Supplementary Figure 2B). Comparisons of the F16–IL2 plus temozolomide group with the other therapeutic groups were performed with the log-rank test and showed significant results (combo *vs* TMZ: *P*<0.002; combo *vs* F16–IL2: *P*<0.002; combo *vs* control: *P*<0.0001).

### Microscopic analysis of effector cell infiltration in subcutaneous and intracranial glioblastoma xenografts, and in normal tissues following treatment

**Figure 3:**
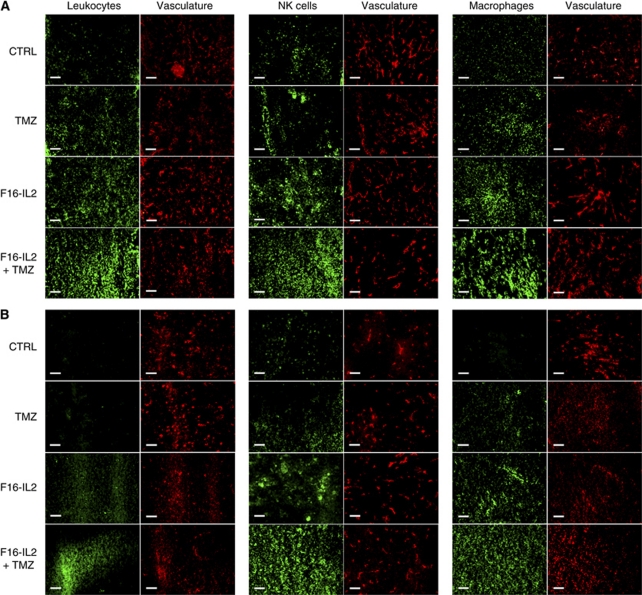
Immunofluorescence analysis of tumour-infiltrating immune cells and of microvascular density in the subcutaneous (**A**) and intracranial (**B**) glioblastoma models, 24 h after the third injection of therapeutic agents. The F16–IL2+temozolomide treatment groups show the largest increase in the infiltration of leukocytes and in particular of natural killer cells and macrophages (serial tissue sections). Scale bars indicate 100 *μ*m.

To assess the infiltration of immune cells into tumours and in normal organs following treatment, subcutaneous and intracranial U87MG human glioblastoma-bearing mice were obtained after three drug administrations, and tumour, liver, and kidney sections were analysed by immunofluorescence. Figure 3 shows representative tumour sections of subcutaneous (A) and intracranial (B) glioblastoma xenografts, stained with antibodies anti-CD45 (a leukocyte-specific marker), anti-asialo GM1 (specific to NK cells), and anti-F4/80 (which recognises macrophages). Vascular structures were costained using an anti-CD31 antibody. The largest increase in infiltration of NK cells and macrophages was observed in combination F16–IL2 plus temozolomide-treated tumours in both subcutaneous and intracranial models (quantification in Supplementary Figure 3), whereas leukocytes were not detected in liver and kidneys of the same animals (Supplementary Figure 4). Pairwise comparisons of the immune cell infiltration into tumours (combo *vs* the other treatments) were calculated using the two-tailed Student's *t*-test and showed significant results (*P*<0.005).

### *Ex vivo* detection of the F16–IL2 fusion protein in subcutaneous and intracranial glioblastoma xenografts following treatment

**Figure 4:**
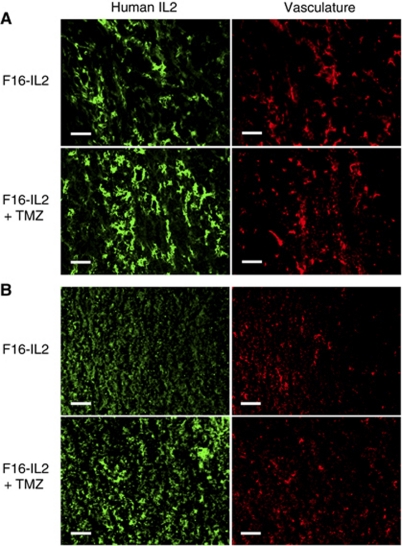
Immunofluorescence analysis of F16–IL2 fusion protein localisation in subcutaneous (**A**) and intracranial (**B**) glioblastoma xenografts, 24 h after the third injection of therapeutic agents (serial tissue sections). Scale bars indicate 100 *μ*m.

We performed an *ex vivo* localisation of the F16–IL2 fusion protein in subcutaneous and intracranial glioblastoma xenografts collected from BALB/c nude mice, which were obtained after three drug administrations of F16–IL2 or the combination of F16–IL2 and temozolomide. The staining for human IL-2 revealed a selective and comparable accumulation of the F16–IL2 immunocytokine around tumour vascular structures in both subcutaneous (A) and intracranial (B) xenografts (Figure 4).

### Microscopic analysis of apoptosis and proliferation in subcutaneous glioblastoma xenografts following treatment

**Figure 5:**
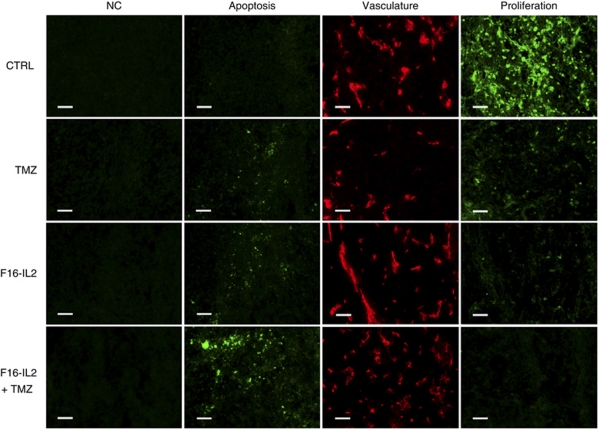
Immunofluorescence analysis of apoptosis and proliferation in subcutaneous glioblastoma xenografts, 24 h after the third injection of therapeutic agents. Results show a clear increase in apoptosis and the complete suppression of proliferation in the combination F16–IL2+temozolomide treatment group. Scale bars indicate 50 *μ*m.

The influence of the different treatments on apoptosis and proliferation in subcutaneous glioblastoma mice was evaluated by immunofluorescence. Figure 5 shows a clear increase in apoptosis and a complete suppression of proliferation in the combination F16–IL2 plus temozolomide treatment group (quantification in Supplementary Figure 6). All pairwise comparisons between the combination and other therapeutic groups showed significant results using the two-tailed Student's *t*-test (apoptosis in combo *vs* all other treatments: *P*<0.001; proliferation in combo *vs* placebo: *P*<0.0001, combo *vs* F16–IL2: *P*<0.009, combo *vs* TMZ: *P*<0.02).

## Discussion

In this article, we have reported on the therapeutic performance of immunocytokine F16–IL2 and of temozolomide, alone and in combination, in subcutaneous and intracranial xenografts of U87MG human glioblastoma.

For both subcutaneous and intracranial U87MG xenografted mice, the combination of F16–IL2 with temozolomide gave the best therapeutic results without additional toxicity, compared with the drugs as single agents (Supplementary Figure 2). In the case of subcutaneous glioblastoma, F16–IL2 potentiated the action of temozolomide, leading to complete tumour eradication in all mice 40 days after beginning the treatment, and to the total remission of animals, which remained tumour free for over 160 days (Figure 2A). In addition, in the intracranial model, the combination treatment was more efficacious, resulting in a 73% decrease in tumour volume 25 days after the start of therapy (Figure 2B), as well as in a longer survival of the animals (Figure 2C).

In both subcutaneous and intracranial xenografts, immunocytokine F16–IL2 promoted the recruitment of immune effector cells into glioblastoma lesions (Figure 3 and Supplementary Figure 3), in analogy to that previously observed in other immunocytokine therapies of mice with solid and haematological malignancies (Carnemolla *et al*, 2002; Halin *et al*, 2002, 2003; Mårlind *et al*, 2008; Schliemann *et al*, 2009). The infiltration of leukocytes was not observed in normal organs from the same mice, thus excluding a nonspecific inflammation caused by F16–IL2 (Supplementary Figure 4). The selective accumulation of F16–IL2 around tumour vascular structures in both subcutaneous and intracranial xenografts (Figure 4), the focal recruitment of immune effector cells (Figure 3), and the therapeutic effect on glioblastoma tumours of the F16–IL2 plus temozolomide combination therapy (Figure 2) support the anticancer role of effector cells stimulated by the immunocytokine.

Our group has previously shown that, surprisingly, the therapeutic effect of IL2-based immunocytokines against murine tumours is identical in immunocompetent and immunocompromised mice, and that NK cells are mainly responsible for the therapeutic action (Carnemolla *et al*, 2002; Halin *et al*, 2003; Ebbinghaus *et al*, 2005). By contrast, immunocytokines based on other cytokines (e.g., tumor necrosis factor, IL12, interferon-*γ*, IL15, and granulocyte macrophage colony-stimulating factor) exhibit a clear dependence on T cells (Borsi *et al*, 2003; Halin *et al*, 2003; Ebbinghaus *et al*, 2005; Kaspar *et al*, 2007). To document further possible mechanisms for the antitumour activity of the immunosuppressive reagent temozolomide in association with the immunostimulatory immunocytokine F16–IL2, we have studied apoptosis and cell proliferation in glioblastoma xenografts after three doses of the four treatments: combination of F16–IL2 with temozolomide, F16–IL2 alone, temozolomide alone, and saline 10% DMSO. Results show a clear increase in apoptosis and complete suppression of proliferation in the combination treatment group, thus supporting a cytostatic plus cytotoxic explanation of therapeutic efficacy (Figure 5 and Supplementary Figure 6).

In our study, F16–IL2 seems to be more effective as a single agent in the intracranial model, whereas the combination of F16–IL2 with temozolomide shows a higher efficacy in the subcutaneous model (Figure 2). We have conducted two independent therapy experiments with the intracranial model (Figure 2B and C), which have confirmed both the therapeutic activity of F16–IL2 on its own and the additive therapeutic benefit observed in combination with temozolomide. There are a number of reasons why the same tumour cells (U87MG) may respond differently to therapy when implanted subcutaneously or orthotopically (in this case, intracranially). One first cause may be that the subcutaneous therapy study started when tumour masses were rather big (300 mm^3^; Figure 2A). In the intracranial study, no magnetic resonance imaging monitoring of lesion size at different time points was possible, but control experiments with the same tumour model suggest that lesions of ∼1–2 mm^3^ were present at day 12 from tumour cell implantation (when our therapy started). Moreover, we have observed in other mouse models of malignancies (melanoma, lymphoma, neuroblastoma, breast, and kidney cancer) that IL2-based immunocytokines may show a minimal therapeutic activity on their own, but strongly synergise with other therapeutic regimens, leading to complete tumour eradication (Schliemann *et al*, 2009; Balza *et al*, 2010; Frey *et al*, 2010; Giavazzi *et al*, manuscript in preparation). In other studies, IL2-based immunocytokines showed a very potent therapeutic activity even when used as a single agent (Carnemolla *et al*, 2002; Menrad and Menssen, 2005; Mårlind *et al*, 2008). Finally, different therapeutic activities in subcutaneous and orthotopic models of glioblastoma have previously been reported by other groups (Friedman *et al*, 1995; Blouw *et al*, 2003; Graf *et al*, 2003; Yamini *et al*, 2007).

Two IL2-based immunocytokines (L19-IL2 and F16–IL2) are currently being investigated in phase I and phase II clinical trials in patients with cancer (Neri and Bicknell, 2005). These fully human immunocytokines can be studied in immunocompetent mouse models of cancer only in an acute setting, as they become immunogenic after a few injections (Carnemolla *et al*, 2002). Furthermore, although L19 reacts with equal affinity with its cognate human and murine antigens (Pini *et al*, 1998; Fattorusso *et al*, 1999), F16 recognises only human and monkey antigens (but not the rodent counterpart; Brack *et al*, 2006; Mårlind *et al*, 2008), thereby avoiding the therapy experiment in a syngeneic tumour model. The predictable effect of IL2-based immunocytokines in immunocompetent patients would be broader and stronger in tumour suppression, because of the involvement of T-cell and B-cell activation. With regard to the role of the blood–brain barrier in brain tumours, we have documented that the tumour-targeting ability of immunocytokine F16–IL2 is comparable in subcutaneous and intracranial mouse xenografts (Figure 4).

The results of this preclinical therapy study may justify the clinical evaluation of F16–IL2 in combination with temozolomide for the treatment of human glioblastoma. F16–IL2 has exhibited an excellent safety profile in cynomolgus monkeys and is currently being studied in two phase Ib clinical trials, in combination with doxorubicin (breast and ovarian cancer) or in combination with paclitaxel (breast and lung cancer).
