# Supplementary figures and images for: Combination of temozolomide with immunocytokine F16–IL2 for the treatment of glioblastoma
Source: Br J Cancer. 2010 Aug 24;103(6):827–36. doi: 10.1038/sj.bjc.6605832 (PMC2966626; doi:10.1038/sj.bjc.6605832)

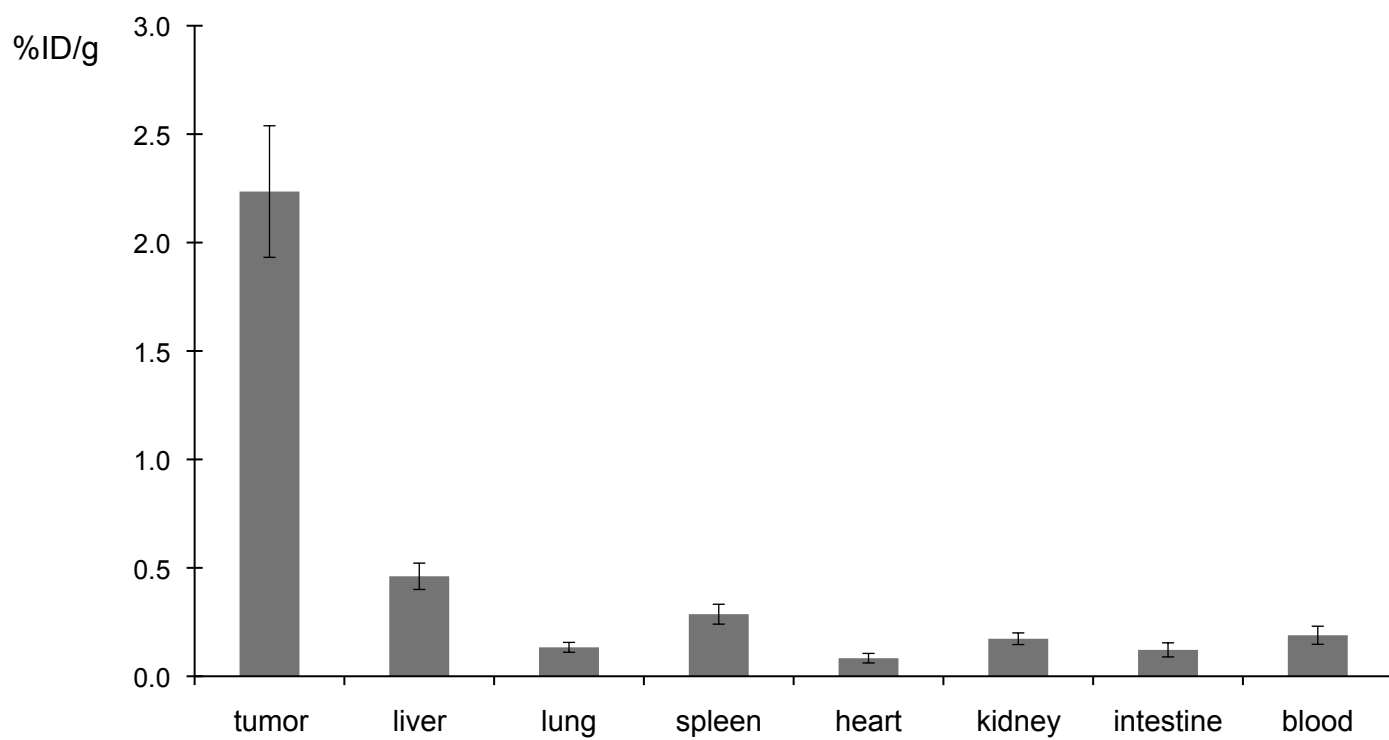

Supplement: Supplementary Figure 1 [file 6605832x1.pdf]

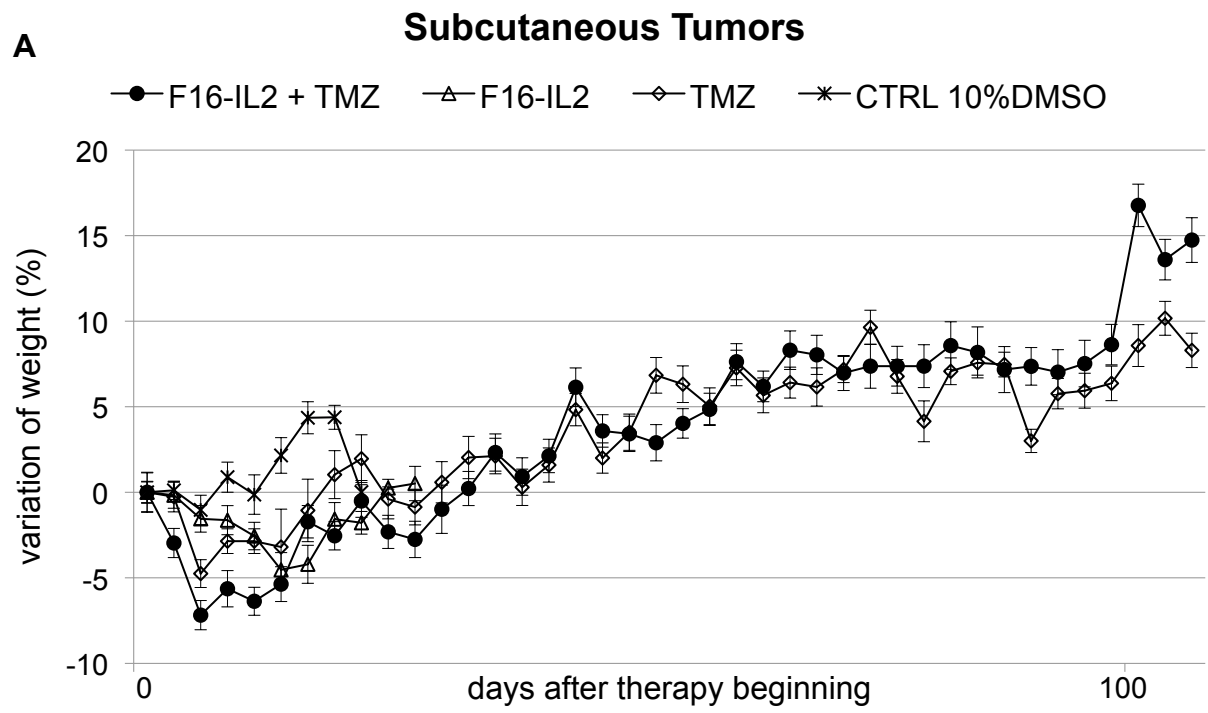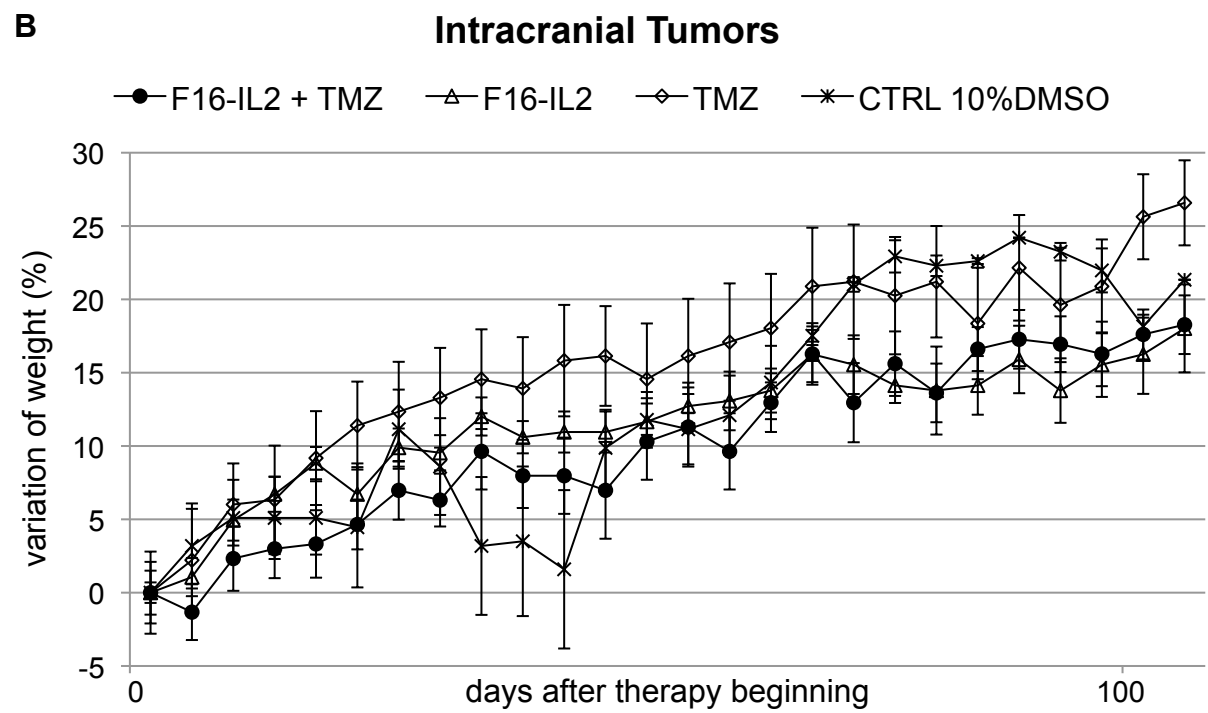

Supplement: Supplementary Figure 2 [file 6605832x2.pdf]

## A Subcutaneous Tumors

■ F16-IL2 + TMZ ■ F16-IL2 ■ TMZ ■ CTRL 10%DMSO

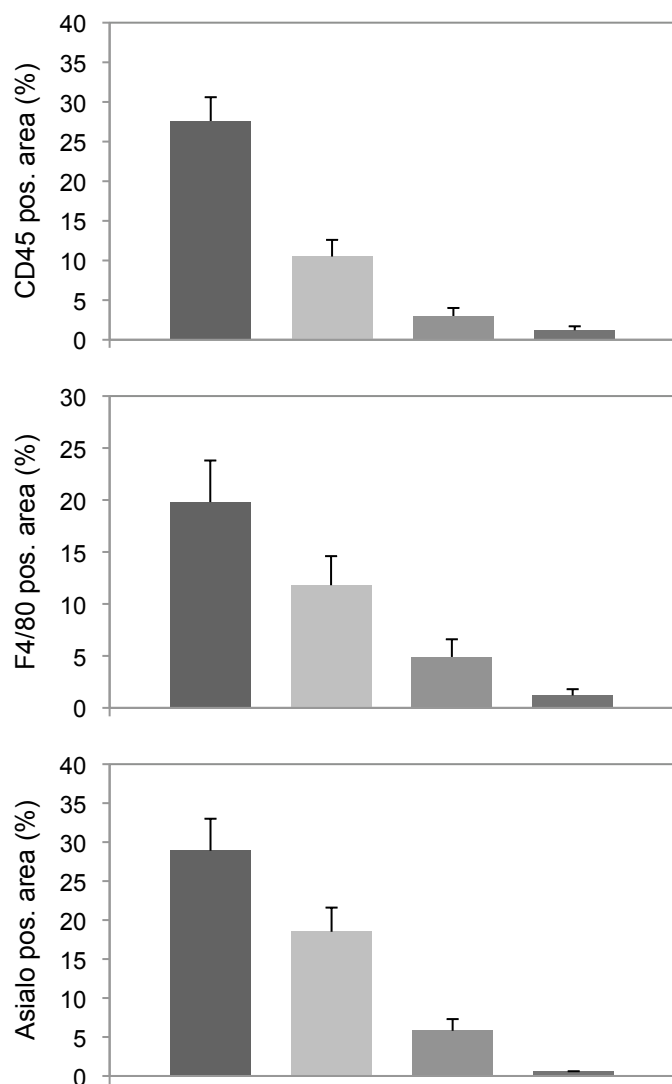

## B Intracranial Tumors

■ F16-IL2 + TMZ ■ F16-IL2 ■ TMZ ■ CTRL 10%DMSO

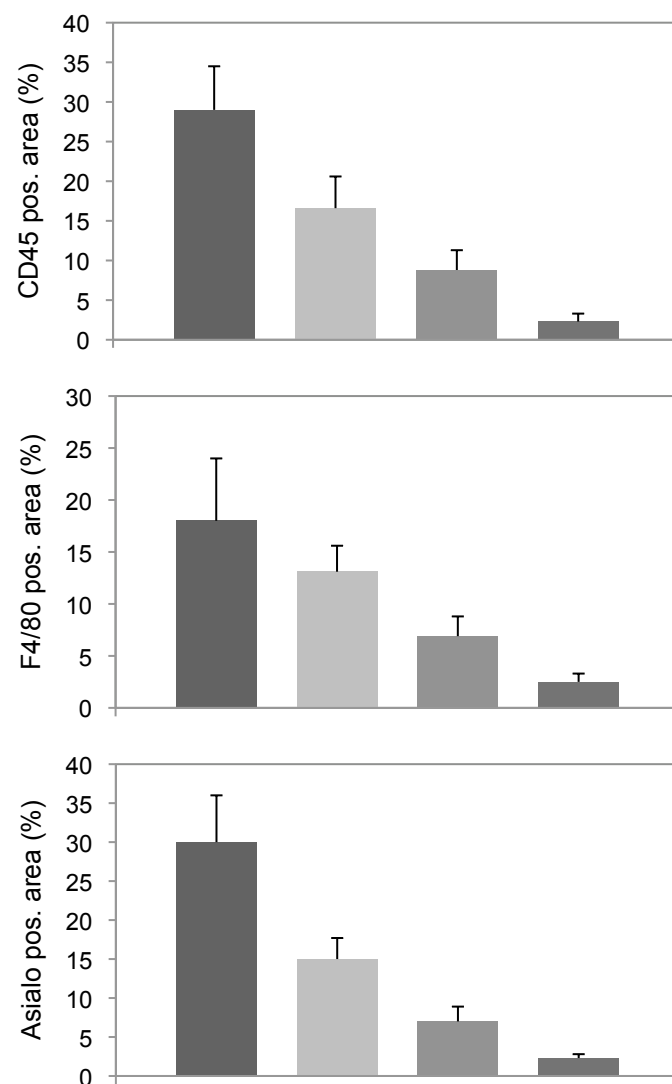

Supplement: Supplementary Figure 3 [file 6605832x3.pdf]

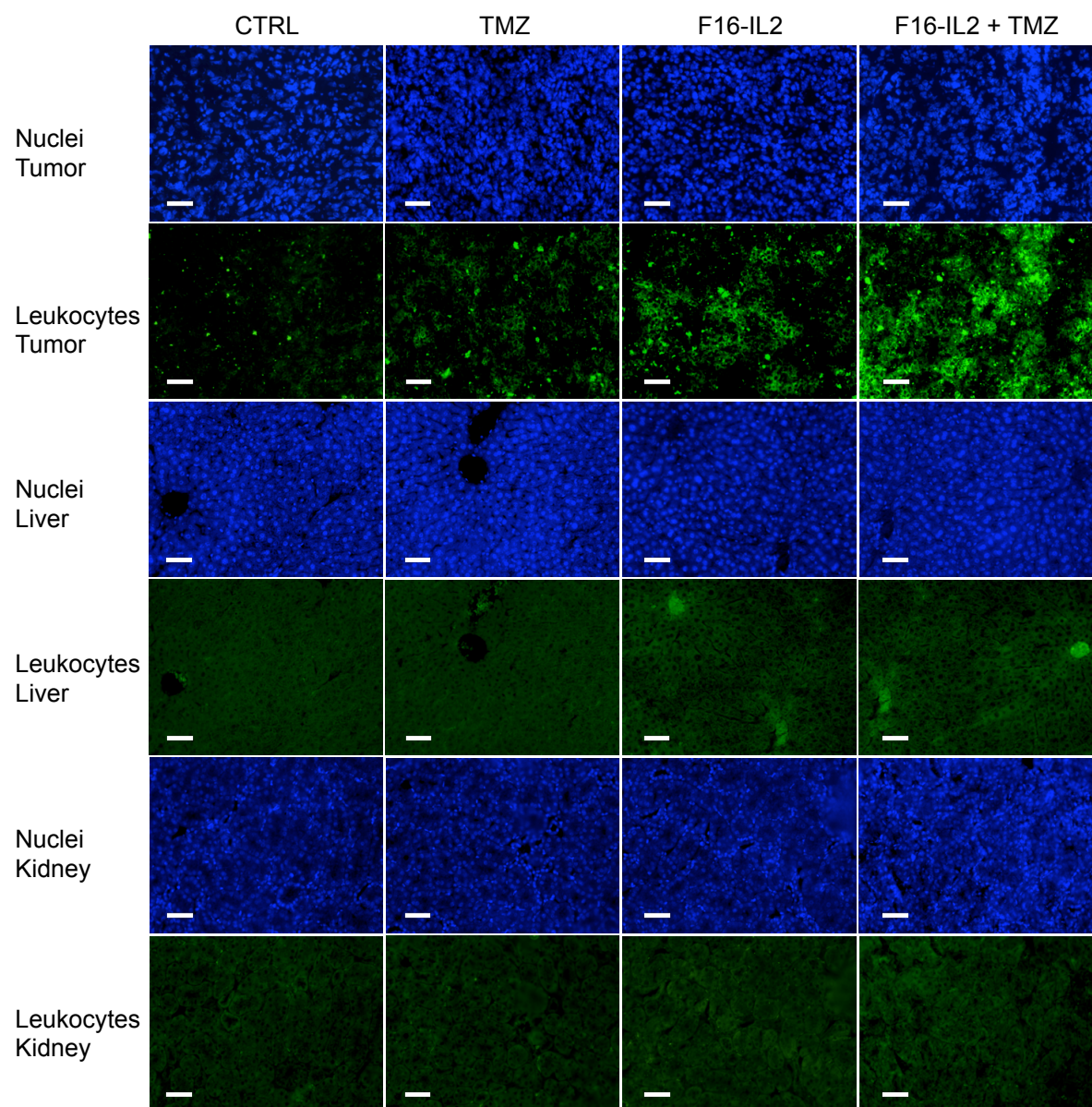

Supplement: Supplementary Figure 4 [file 6605832x4.pdf]

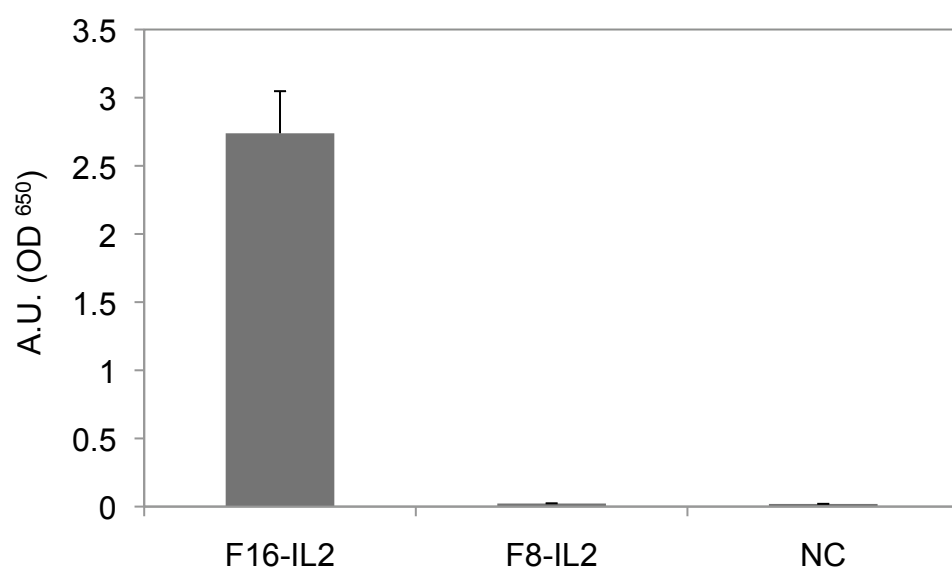

Supplement: Supplementary Figure 5 [file 6605832x5.pdf]

■ F16-IL2 + TMZ ■ F16-IL2 ■ TMZ ■ CTRL 10%DMSO

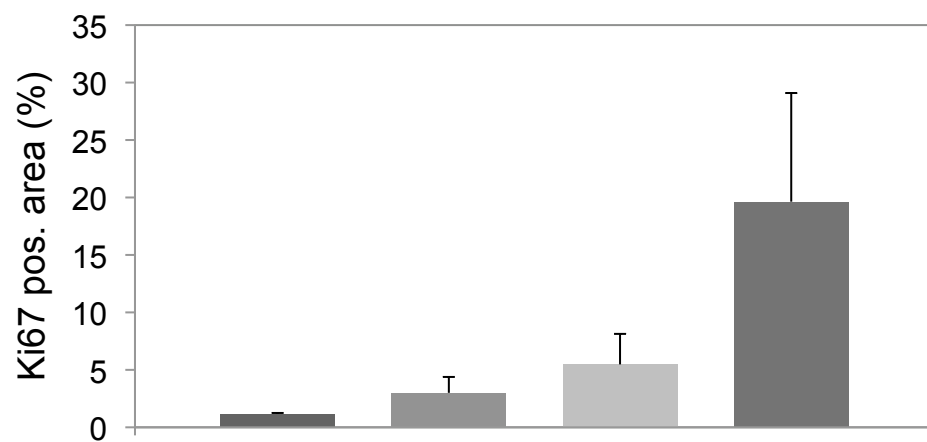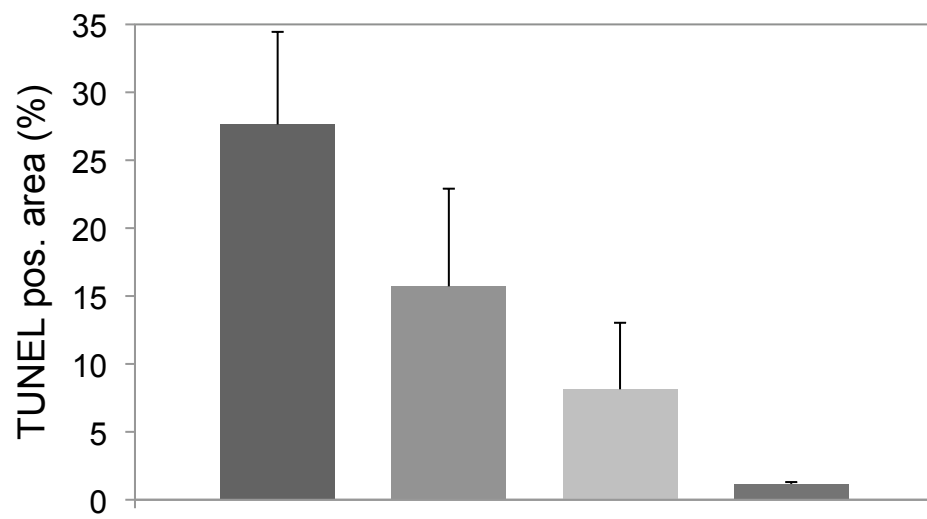

Supplement: Supplementary Figure 6 [file 6605832x6.pdf]
