# Supplementary material for: Combination of temozolomide with immunocytokine F16–IL2 for the treatment of glioblastoma
Source: Br J Cancer. 2010 Aug 24;103(6):827–36. doi: 10.1038/sj.bjc.6605832 (PMC2966626; doi:10.1038/sj.bjc.6605832)
Supplement: Supplementary Figure Legends [file 6605832x7.doc]

SUPPLEMENTARY FIGURE LEGENDS

**Suppl. Fig. 1:** Quantitative biodistribution analysis of the radiolabeled F16-IL2 fusion protein in nude mice with U87MG subcutaneous xenografts. A preferential accumulation in the tumor was observed 24h after injection (2.3% ID/g), with a tumor-to-blood ratio of 11.5 and with excellent tumor-to-organ ratios.

**Suppl. Fig. 2:** Analysis of the variation of weight of mice with subcutaneous **(A)** and intracranial **(B)** U87MG xenografts during a five-administration treatment with F16-IL2 + temozolomide, F16-IL2 alone, temozolomide alone, or the placebo (saline 10% DMSO).

**Suppl. Fig. 3:** Quantitative analysis of immune cells infiltration into subcutaneous **(A)** and intracranial **(B)** U87MG human glioblastoma xenografts, after a three-administration treatment of F16-IL2 + temozolomide, F16-IL2 alone, temozolomide alone, or the placebo. Pairwise comparisons (combo *vs.* the other treatments) were calculated using the Student’s *t*-test and resulted significant (*P* < 0.005).

**Suppl. Fig. 4:** Immunofluorescence analysis of effector cell infiltration in tumors and in kidneys and liver of subcutaneous U87MG xenografted mice, 24h after the third injection of the therapeutic agents. No infiltration is detected in normal tissues, whereas leucocytes appear to penetrate tumors of mice treated with F16-IL2 + temozolomide. Scale bars indicate 100 μm.

**Suppl. Fig. 5:** The specific binding of the rat anti-human IL-2 antibody to the F16-IL2 immunocytokine was validated by ELISA. We loaded the biotinylated tenascin-C A1 antigen (10^-6^ M) on streptavidin wells (A-H), followed by 5 μg/ml of F16-IL2 in PBS 2% milk (wells A-C), and 5 μg/ml of F8-IL2 (wells D-F) as irrelevant antibody; wells G-H served as negative control without primary antibody. The rat anti-human IL-2 antibody (eBioscience Inc, San Diego, USA; diluted 1:1000) was used as secondary antibody (wells A-H), followed by the goat anti-rat IgG-HRP (eBioscience Inc, San Diego, USA; diluted 1:1000), the POD substrate (Roche Diagnostic, Rotkreuz, Switzerland), and the H_2_SO_4_ 1M solution to complete the reaction. Error bars indicate standard deviation.

**Suppl. Fig. 6:** Quantitative analysis of proliferation (Ki67) and apoptosis (TUNEL assay) in subcutaneous U87MG xenografts after a three-administration treatment of F16-IL2 + temozolomide, F16-IL2 alone, temozolomide alone, or the placebo. Pairwise comparisons were calculated using the Student’s *t*-test and resulted significant (apoptosis in combo *vs.* all other treatments: *P* < 0.001; proliferation in combo *vs.* placebo: *P* < 0.0001, combo *vs.* F16-IL2: *P* < 0.009, combo vs. TMZ: *P* < 0.02).
